# Weak Knees: A Case of Atorvastatin-induced Dermatomyositis

**DOI:** 10.7759/cureus.7387

**Published:** 2020-03-24

**Authors:** Edgar Gutierrez, Mehdi Faraji, Lauren Pacheco

**Affiliations:** 1 Radiology, Brookwood Baptist Medical Center, Birmingham, USA; 2 Radiology, Louisiana State University Health Sciences Center Shreveport, Shreveport, USA; 3 Internal Medicine, Brookwood Baptist Medical Center, Birmingham, USA

**Keywords:** statin, dermatomyositis, weakness, myopathy

## Abstract

HMG-CoA reductase inhibitors (statins) are one of the most widely used medications in the primary care setting, and like any medications they have many side effects. The common ones include myalgias and rare ones include dermatomyositis. Here we present the case of atorvastatin-induced dermatomyositis with an unfortunate progression. This mandates a low threshold for first contact doctors to screen their patients for new-onset muscle weakness and rash after starting a statin recently, like our patient who had started atorvastatin several months before. This case adds to the previously reported cases and provides further evidence for statins being triggers of immune-mediated disease. The appropriate management of this condition requires a collaborative effort involving clinical judgment, laboratory testing, and imaging.

## Introduction

Statins are widely used lipid-lowering medications for the prevention of cardiovascular disease. Statins are generally well tolerated and safe, making them an excellent choice in therapy. However, there are common instances of statin-induced myalgias and rare instances of moderate-to-severe cases of statin-induced myopathies [1-6]. Statin-induced myopathy consists of a spectrum of disease conditions including polymyositis (PM), dermatomyositis (DM), and immune-mediated necrotizing myositis (IMNM). DM has been known to be an iatrogenic, autoimmune, and a paraneoplastic syndrome. Several previous cases of statin-induced DM have been reported in the literature with courses ranging from relatively benign to fatal [1]. We present the case of a patient with atorvastatin-induced DM. 

## Case presentation

**Figure 1:**
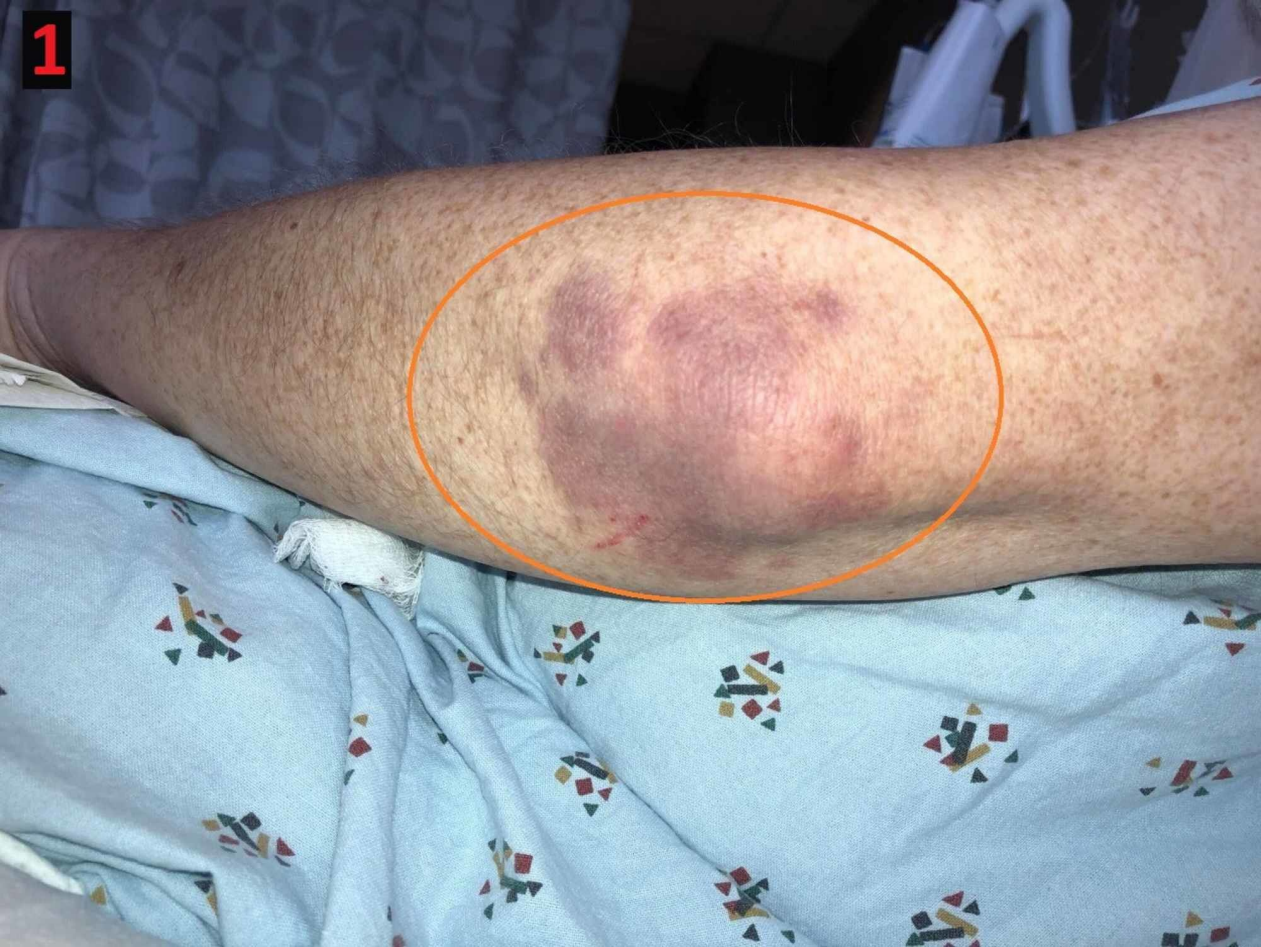
Gottron's sign Erythematous patch overlying the extensor surface of the left elbow (orange circle)

**Figure 2:**
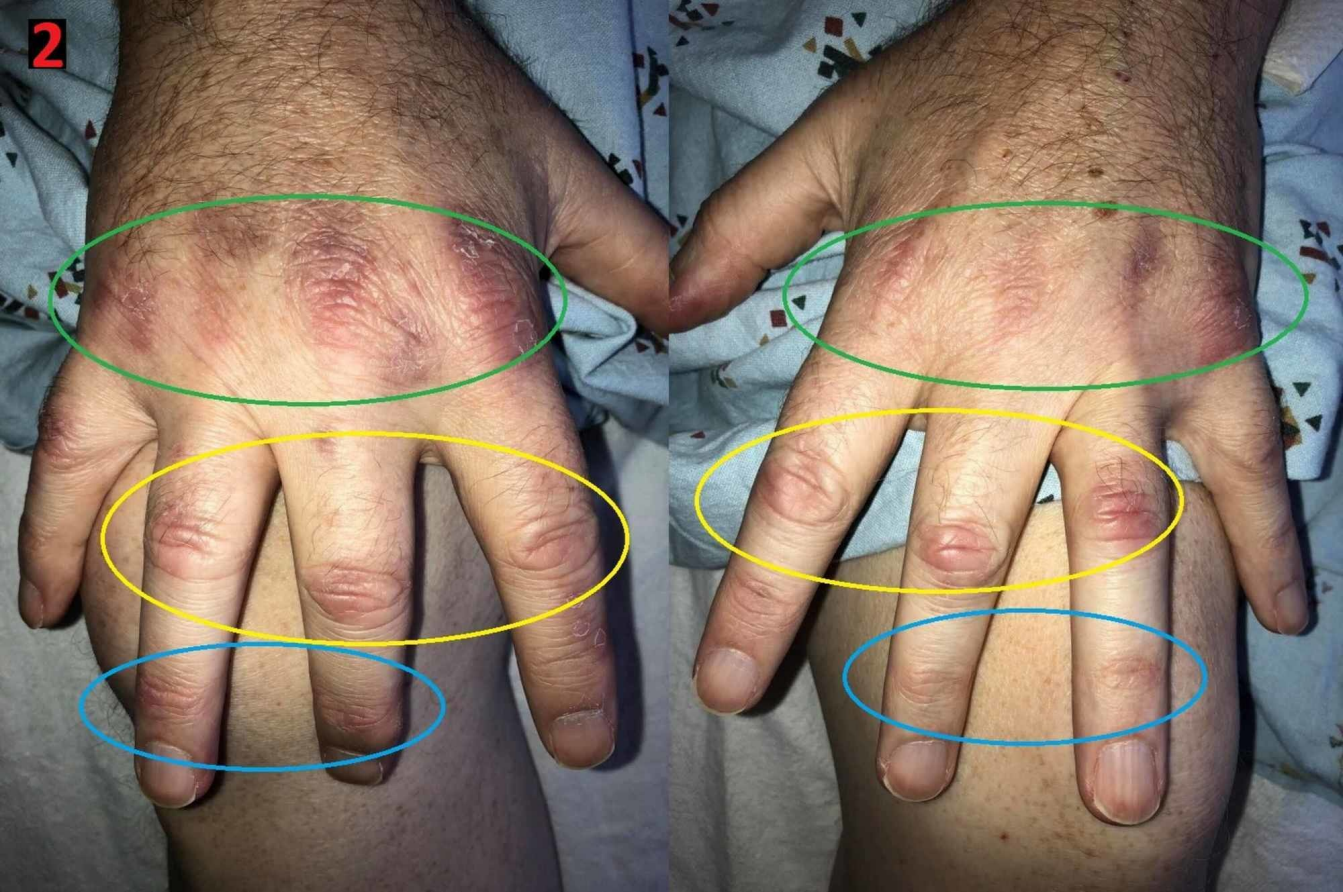
Gottron’s papules Erythematous papules/plaques seen on the extensor surfaces of the metacarpophalangeal joints (green ovals), proximal interphalangeal joints (yellow ovals), and distal interphalangeal joints (blue ovals) on the right and left hands

A 49-year-old Caucasian male presents with a one-month history of progressively worsening dysphagia and proximal muscle weakness. Comorbidities include diabetes mellitus type 2 and hyperlipidemia. Home medications include semaglutide and atorvastatin. Two weeks prior to presentation, he noticed diffuse erythematous, circular plaques that appeared abruptly on his extremities, torso, back, and face. His primary care doctor discontinued the statin and referred him to gastroenterology and dermatology for dysphagia and rash, respectively. He underwent esophagogastroduodenoscopy for dysphagia showing esophageal stricture and was given a topical steroid cream for his rash. The patient was placed on oral prednisone therapy one week prior to arrival without symptom improvement. Due to worsening symptoms, he presented to our emergency department for further evaluation. Physical examination showed that the patient had 3/5 muscle strength at the shoulders and hips bilaterally. Other findings included heliotrope rash and Gottron’s papules (Figures 1, 2).

Initial laboratory studies revealed elevated white blood cells at 29 (x10^9 cells/L), creatine kinase (CK, 6,000 mg/dL), myoglobin (12,000 ng/mL), aspartate aminotransferase (430 U/L), and alanine transaminase (700 U/L). Notable negative/normal laboratory values include C3, C4, antinuclear antibody, rheumatoid factor, hepatitis panel, antineutrophil cytoplasmic antibodies, anti-tissue transglutaminase (IgA/IgG), and myositis panel (including anti-Mi-2 and anti-Jo-1 autoantibodies).

**Figure 3:**
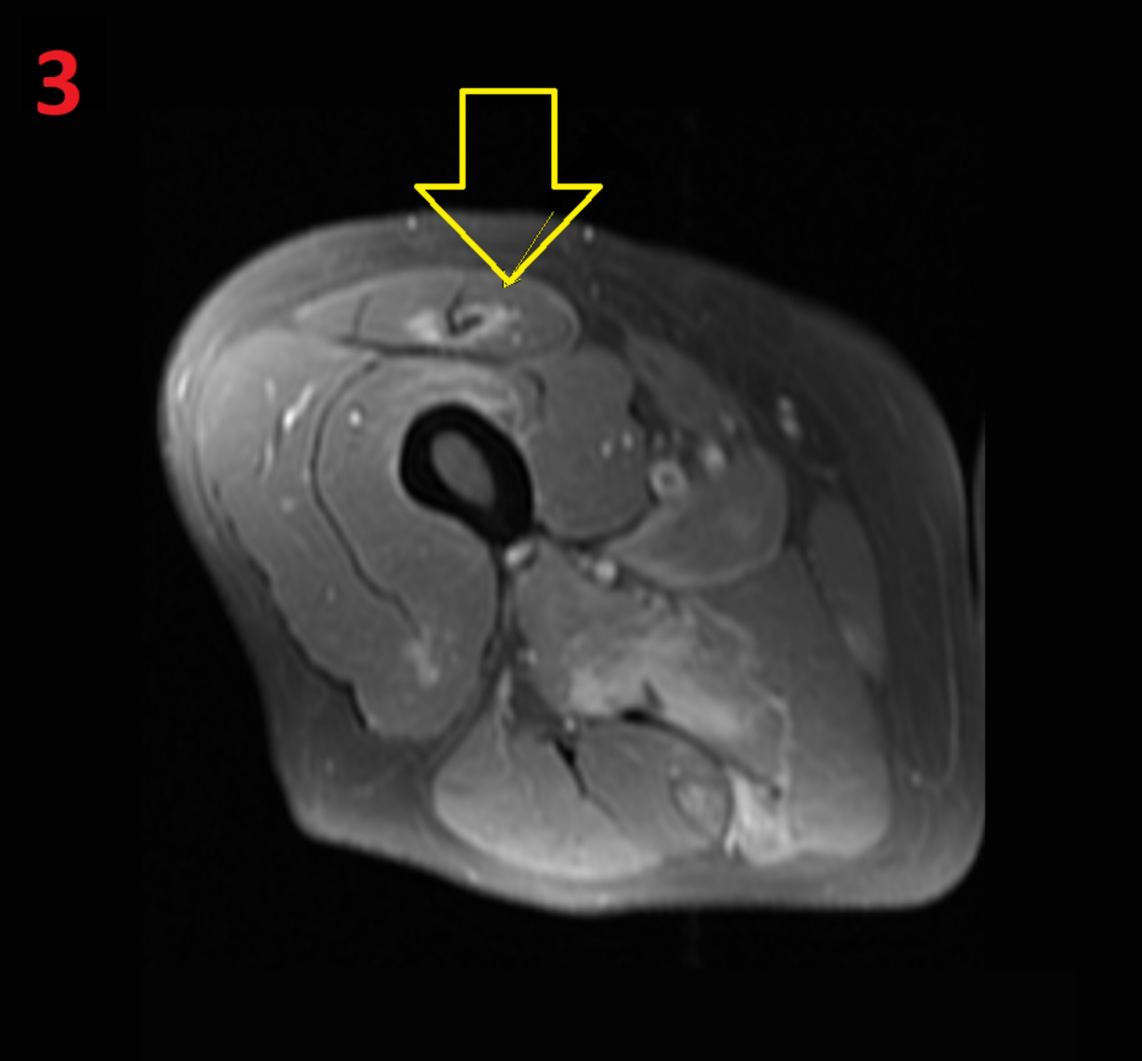
MRI of the left hip Fat-saturated post-contrast T1-weighted MRI of the left hip, showing an edematous signal within the rectus femoris (yellow arrow)

**Figure 4:**
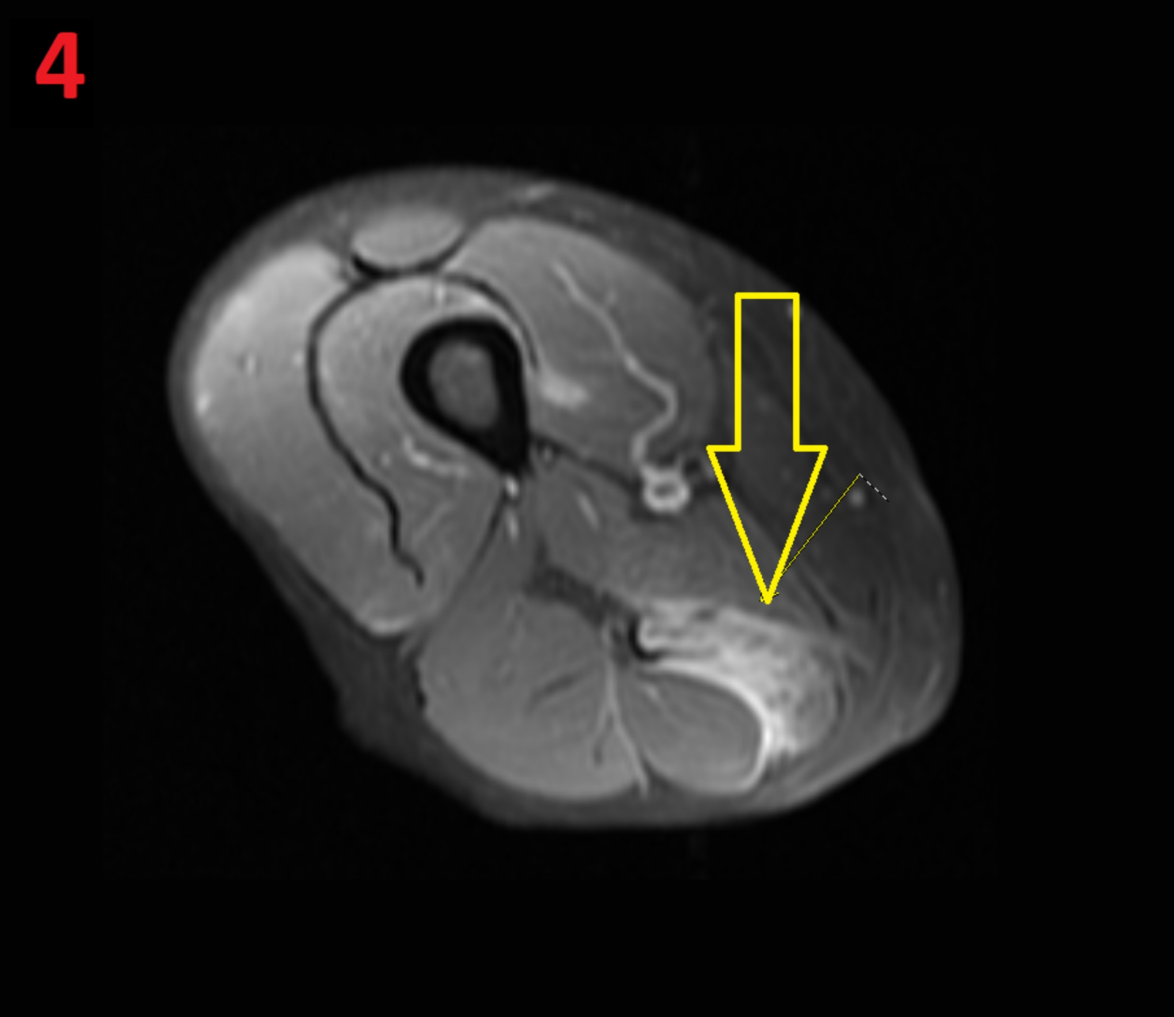
MRI of the left hip Fat-saturated post-contrast T1-weighted MRI of the left hip showing areas of intramuscular fat stranding and edematous signal within the semimembranosus muscles (yellow arrow)

During his hospital course, he had persistent tachycardia and leukocytosis prompting a cardiac and infectious workup which came back normal. His treatment included one week of high-dose intravenous steroids and 20 mg oral prednisone thereafter, and a course of intravenous immunoglobulin. Despite treatment, he continued to have a rash with progressive proximal weakness and dysphagia as well as the development of head drop (neck muscle weakness). Malignancy workup was performed with contrast-enhanced CT scan of the head, chest, abdomen, and pelvis which were normal. Electromyography showed non-specific findings of myositis. MRI of the left lower extremity showed bilateral diffuse muscle enhancement on T1-weighted imaging with extensive muscle edema and evidence of fat stranding was seen (Figures 3, 4).

Muscle biopsy of the left vastus lateralis was consistent with necrotizing myopathy. At this point in time, the diagnosis of DM secondary to statin use was made. The patient was placed on prednisone 20 mg daily. Over the course of the next few months, the patient’s clinical course continued to deteriorate and he eventually required a percutaneous endoscopic gastrostomy tube and a wheelchair. He underwent subsequent readmission with high-dose intravenous steroids followed by high-dose oral steroid taper over the next several months. In the outpatient neurology setting, he was treated with regular rituximab infusions. Eight months after symptom onset, the patient was using a cane to walk and the severity of the skin rash was no better.

## Discussion

This patient presented with classic features of DM, including proximal and symmetrical muscle weakness, Gottron’s papules, and heliotrope rash. MRI of muscle involvement showed characteristic findings of DM, including diffuse muscle enhancement on T1-weighted imaging suggesting fatty infiltration and diffuse bilateral muscle edema [1]. This patient had been on statin therapy for several months before presentation which is consistent with the timeline of most statin-induced myopathies [4]. Several statin-induced DM cases have been reported in previous literature [1-6].

Statin-induced DM usually presents after months to years of previous statin therapy but can also present in the first few days [1]. Our patient had been on statin therapy for five months prior to symptom onset. This report presents a rare case of statin-induced DM with prominent necrosis seen on muscle biopsy. One could argue that this case presents a diagnosis of IMNM based on the muscle biopsy findings alone. However, IMNM presents with muscle involvement alone and would not explain the liver and skin findings in this patient, which can be seen in DM [2-4]. Additionally, the CK level findings in IMNM tend to be in excess of 10,000 U/L compared to our patient with CK levels around 5,000 U/L. The test for anti-HMGCR antibodies can be helpful in excluding the diagnosis of IMNM. However, the test for anti-HMGCR antibodies has a low positive predictive value making this test non-diagnostic. Hence, clinical correlation would still be required to make a diagnosis of IMNM [4].

This case adds to the previously reported cases and provides further evidence for statins being triggers of immune-mediated disease, although the exact mechanism is poorly understood. This case provides further evidence, and emphasizes the need for vigilance and close follow-up when assessing patients on statin therapy who present with proximal muscle weakness. The benefit of knowing the particular type of myopathy whether it be PM/DM/IMNM is of questionable clinical significance as there are no current treatment guidelines that would distinguish them at of the time of this report. However, most patients presenting with PM/DM-like symptoms undergo cancer screening, as to rule out a paraneoplastic syndrome.

## Conclusions

Statins are one of the most common medications that patients are on, and every good medical student knows about statin-induced myalgias, but statin-induced DM is also known to occur. DM presents with symmetrical muscle weakness, Gottron’s papules, and heliotrope rash with MRI finding of muscle edema and fatty infiltration. Muscle biopsy findings will include muscle necrosis. Patients on statin therapy presenting with proximal muscle weakness should have immediate intervention with cessation of the statin and aggressive immune suppression as complications of statin-induced DM can result in significant disability and sometimes even death. Our case report describes this unusual scenario with an unfortunate outcome; thus, it is important that the primary care, dermatological, and radiological communities are aware of these findings and complications from the disease. Hence, the appropriate management of this condition requires a collaborative effort involving clinical judgment, laboratory testing, and imaging.
